# Utility of Circulating MicroRNAs as Clinical Biomarkers for Cardiovascular Diseases

**DOI:** 10.1155/2015/821823

**Published:** 2015-02-01

**Authors:** Altaf A. Kondkar, Khaled K. Abu-Amero

**Affiliations:** ^1^Ophthalmic Genetics Laboratory, Department of Ophthalmology, College of Medicine, King Saud University, Riyadh 11411, Saudi Arabia; ^2^Glaucoma Research Chair, Department of Ophthalmology, College of Medicine, King Saud University, Riyadh 11411, Saudi Arabia; ^3^Department of Ophthalmology, College of Medicine, University of Florida, Jacksonville, FL 32209, USA

## Abstract

MicroRNAs (miRNAs) are small noncoding RNA molecules that regulate gene and protein expression by translational repression and/or mRNA degradation. miRNAs are implicated in the pathogenesis of various cardiovascular diseases and have become potential targets for therapeutic intervention. Their stability and presence in variety of readily accessible cell types including whole blood, serum, plasma, and other body fluids render them as potential source of a clinical biomarker. This review provides a brief overview of miRNA biogenesis and function, the diagnostic potential of circulating extracellular miRNA and their specific role in vivo in various cardiovascular settings, and their future perspective as clinical biomarkers. It is clearly evident from experimental studies that miRNAs are responsible for the regulation of several biological functions and alterations in cardiovascular diseases. Current data supports the concept of using circulating miRNAs as a biomarker in cardiovascular disease. It remains to be seen, however, whether circulating miRNAs can fulfil this role to improve risk and severity prediction.

## 1. Introduction

Cardiovascular diseases (CVD) are the leading cause of death in both the developed and developing countries. Epidemiological studies have identified various modifiable risk factors that negatively affect cardiovascular health. Understanding the complex molecular aetiology of the disease continues to be a major focus of interest of multiple research groups. Identification of factors that may significantly reduce the patient mortality rates still remains a challenge. Early diagnosis of acute coronary syndrome (ACS) leading to myocardial infarction (MI) and unstable angina is highly essential for patient management and treatment to reduce cardiovascular mortality [1, 2]. Proteins such as cardiac troponin (cTn), creatine-kinase myocardial band isoform (CK-MB), brain natriuretic peptide (BNP), and its N-terminal prohormone (NT-proBNP) have been utilized as biomarkers to detect and monitor myocardial damage [3]. With the increased specificity and sensitivity of cTn, CK-MB is no longer utilized in the clinical settings [4].

cTnI and cTnT are the most preferred biomarkers for the diagnosis of AMI [4]. cTn levels rise as early as 3 h after the onset of chest pain and remain elevated for up to 10 days after AMI. Interestingly, cTn levels correlate with the infarct size and prognosis [5]. Although cTns are excellent biomarkers they lack the ability to discriminate ACS and MI from other clinical syndromes [6]. There exist considerable variations in the specificity and sensitivity of the assays. The high-sensitivity cTnT (hs-cTnT) assay at 99th percentile (≥14 ng/L) shows a low positive predictive value that limits the rule in and might result in a substantially higher number of false-positive findings, especially when the pretest probability of ACS is low [7]. In addition, there exists a relative “delayed” release time of troponin [8]. Undoubtedly, there is a need for earlier clinical biomarker to differentiate between cardiac injury from ACS and noncardiac conditions and assess the risk of cardiovascular events. MicroRNAs (miRNAs) have been recently implicated in various cardiovascular diseases. This review provides an overview of basic biology and function of miRNAs highlighting the in vivo contribution of specific miRNAs in cardiovascular pathology with emphasis on the potential clinical utility of circulating extracellular miRNAs as biomarkers in various cardiovascular settings.

## 2. miRNA Biology

miRNAs regulate gene expression posttranscriptionally by degrading messenger RNA (mRNA) targets and/or by blocking their translation [9, 10]. Each miRNA can target multiple mRNAs and regulate ~60% of mammalian protein-coding genes [11, 12]. miRNAs have diverse functions in the regulation of several key biological and cellular processes including differentiation, development, growth, proliferation, and apoptosis and thus have the potential to modulate complex physiological or disease phenotypes [13–15]. Currently, at least 2,000 unique mature human miRNAs are documented in the miRBase database [16].

## 3. Biogenesis, Secretion, and Biological Functions of Circulating miRNA

**Figure 1:**
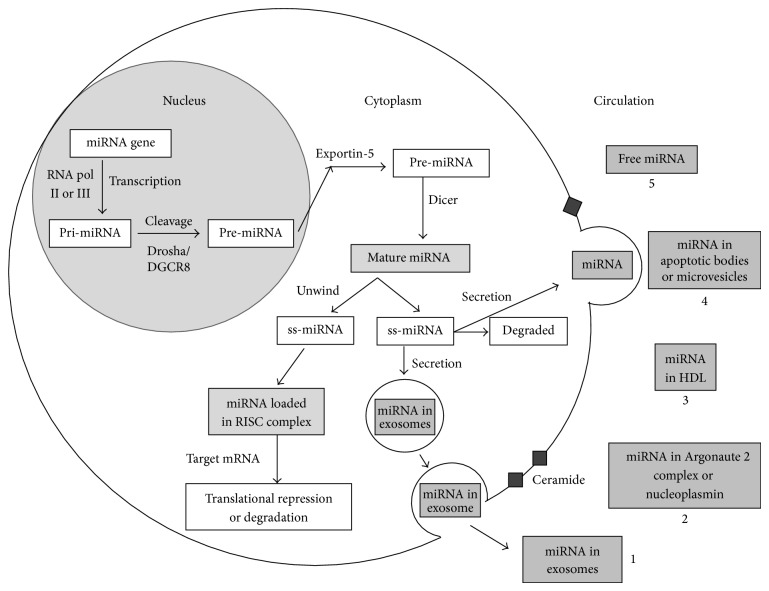
A simplified flow chart depicting the steps involved in intracellular biogenesis of miRNA and their secretion in circulation. It is hypothesized that the mature miRNA can be secreted outside the cell via (1) exosomes when multivesicular body fuses with the cell membrane, (2) miRNA-protein complexes (Argonaute 2, nucleoplasmin), (3) HDL-miRNA complexes, (4) apoptotic bodies or microvesicles through interaction with membrane proteins, and (5) free miRNAs by natural spill or as by-products of dead cells. Various tissues and blood cells can contribute to the circulating miRNA pool. In otherwise undetectable levels in circulation, cardiac-specific miRNAs are released into the bloodstream in response to injury, such as AMI, and may serve as biomarkers for cardiovascular diseases.

The flow chart in Figure 1 depicts the different steps involved in the biogenesis and secretion of circulating extracellular miRNA. Most of the miRNA genes are intergenic [17] or are located in the introns of the protein-coding genes [18]. The primary miRNA (pri-miRNA) is transcribed from miRNA genes in the nucleus, which is subsequently processed into a precursor or pre-miRNA by RNase III enzyme, Drosha, and DCGR8 [19]. The pre-miRNA molecule is transported into the cytoplasm by exportin-5 [20], where they are further processed by Dicer, another RNase III endonuclease, into the mature single-stranded miRNAs, and subsequently incorporated into the RNA-induced silencing complex [21], to regulate targeted gene expression [22, 23]. More detailed information on the biogenesis and function of miRNAs can be reviewed elsewhere [9, 21].

A number of investigators have demonstrated the existence of mature miRNAs in extracellular fluid, for example, blood, plasma, and serum [24, 25]. Although precise mechanism(s) for secretion of miRNA from cells is largely unknown, ceramide-dependent secretory machinery and passive leakage from apoptotic cells have been proposed [26, 27]. Interestingly, circulating miRNAs display remarkable stability even under harsh conditions, including endogenous RNase activity, multiple freeze-thaw cycles, boiling, high and low pH, and long-term storage at room temperature, which renders them extremely suitable for use of blood-based biomarkers [24, 25]. Taken together these observations suggest existence of mechanism(s) that shield circulating miRNAs from degradation outside the cell.

Several theories have been described to explain miRNA transport and stability outside the cell. Detection of miRNAs in peripheral blood microvesicles [28] led to the theory that extracellular miRNA is protected by encapsulation into membrane vesicles. Microvesicles from the plasma of patients with atherosclerosis containing higher levels of miR-150 were shown to effectively regulate migration and expression of miR-150 target gene* c-MYB*, demonstrating that circulating miRNA can mediate intercellular communication and regulate gene expression in an intracellular manner [29]. Another type of membranous microvesicles is apoptotic bodies. Endothelial-cell derived apoptotic bodies are enriched in miR-126 and their delivery in apolipoprotein E^−/−^ mice reduced atherosclerosis via recruitment of progenitor cells indicating that these apoptotic bodies can also contain miRNAs and secrete them to neighbouring cell [27]. miRNAs are also actively secreted in exosomes when a multivesicular body fuses with the plasma membrane and release the exosomes in a ceramide-dependent process [30]. Besides microvesicles, the majority of circulating miRNAs exist in a nonvesicle form, in protein-bound complexes. Nucleoplasmin, a nuclear protein implicated in ribosomal export, was found to be bound to some miRNAs but not all [30]. A second protein, Argonaute 2, derived from human plasma was also found to contain specific miRNAs (e.g., miR-92a) [31]. Additionally, Vickers et al. demonstrated high-density lipoprotein (HDL) isolated from patients with familial hypercholesterolemia to be enriched in miRNAs (e.g., miR-223, miR-105, and miR-106a) suggesting lipid proteins as carrier molecules for miRNAs [32]. Circulating miRNA may be physiologically active and play a critical role in cell-to-cell communication via paracrine routes [33, 34]. However, which of these modes of secretion plays a role under physiological conditions and in diseased states remains to be elucidated.

## 4. Circulating miRNA as Biomarkers

Circulating, cell-free nucleic acids from sera and other body fluids have the potential to serve as biomarkers for early detection of several human diseases [35]. An ideal biomarker can be considered one that is tissue specific, disease (and stage) specific, functional with long half-life, easily detectable and quantifiable for point-of-care laboratory testing, and readily accessible using less invasive procedures. miRNAs seem to fulfil these characteristics. miRNAs are known to be tissue- or cell specific and correlate with disease phenotype. Circulating miRNAs have been reported in whole blood, peripheral blood mononuclear cells (PBMCs), platelets, serum, plasma, and other body fluids [25] and can be detected with high sensitivity and specificity using real-time PCR, deep sequencing, and microarray. miRNAs are stable in circulation [32, 36]. Plasma miRNAs were found to be stable for 24 h at room temperature and through 8 freeze-thaw cycles [37]. In recent years, circulating miRNAs have generated great interest and have been investigated as a source of novel clinical biomarkers in several human diseases [38–41].

## 5. Circulating miRNAs as Biomarkers in Cardiovascular Disease

miRNAs play a critical role in cardiac development and pathological processes including AMI, arrhythmias, hypertrophy, heart failure, and atherosclerosis [42]. Over 200 miRNAs have been detected in the heart. miRNAs such as miR-1, let-7, miR-133, miR-126-3p, miR-30c, and miR-26a were found to be predominant in the cardiac muscles and miR-145, let-7, miR-125b, miR-125a, miR-23, and miR-143 in the arterial smooth muscles [43, 44]. Numerous studies have proposed circulating miRNA content to serve as useful marker of cell activation and tissue injury in response to cardiovascular disease and its associated risk factors (Table 1).

## 6. Acute Myocardial Infarction

In cardiovascular diseases, AMI is the ideal scenario to establish the potential role of circulating miRNA as biomarkers. Given that MI results in release of cardiac proteins such as cTn into the bloodstream, it can be hypothesized that AMI also leads to release cardiac-specific miRNAs in circulation.

miR-1 is abundantly expressed in the cardiac and skeletal muscles. Studies have shown that miR-1 levels are increased in both animal models and patients with AMI. miR-1 is encoded by 2 genes* MIR-1-1* and* MIR-1-2* encoded by two distinct chromosomes 18 and 20, respectively. miR-1 plays a crucial role in cardiac development and muscle differentiation. Homozygous deletion of* MIR-1* gene (*MIR-1-2*) in mice resulted in late embryonic lethality and sudden cardiac death among survivors, the majority of which were due to arrhythmias, indicating that miR-1 may regulate cardiac arrhythmogenicity by targeting the gap junction protein, connexin (*GJA1*), and potassium ion channel (*KCNJ2*) [45, 46].

In a murine AMI model, circulating miR-1 levels rapidly increased 1 hr after AMI, peaking 200-fold at 6 hrs and returning to baseline levels 3 days after AMI. The circulating levels showed strong positive correlation with size of infarct [47]. In a mouse AMI model, however, miR-1 levels began to rise 6 hrs after AMI, suggesting a species specific time course release [48]. In addition, levels of miR-1 and miR-133 after induced AMI in adult pigs showed strong correlation with renal glomerular filtration rate, indicating the effect renal elimination can have on circulating miR levels [49].

In a small study of 31 AMI patients, Cheng et al. reported nearly 100-fold increase in serum miR-1 levels 6 hrs after AMI. The levels showed positive correlation with serum CK-MB levels [50]. The findings were corroborated in another study by Ai et al. in 159 patients with and without AMI. Plasma miR-1 levels were found to be significantly high in the AMI patients independent of age, gender, diabetes, and other well-established AMI biomarkers and the levels correlated well with QRS complex [51]. However, no correlation was reported between miR-1 and ST-segment or cTnI and CK-MB, highlighting the difference in the pattern of release of cardiac proteins and miRNA during the progression of myocardial necrosis.

miR-133 is expressed in cardiac, skeletal, and smooth muscle cells of the cardiovascular system [52]. The miR-133 family contains 3 miRNA genes,* MIR-133a-1*,* MIR-133a-2*, and* MIR-133b*, each of which is cotranscribed bicistronically with* MIR-1-2*,* MIR-1-1*, and* MIR-206*, respectively. Genetic deletion of both* MIR-133a* genes in mice results in embryonic lethality accompanied with inhibition of cardiomyocytes proliferation, apoptosis, and aberrant expression of smooth muscle genes in the heart [53]. miR-133 has been shown to control vascular smooth muscle cell phenotypic switch, thus contributing to the progression of atherosclerosis [54].

In an investigation by Wang et al. in 33 Chinese patients with AMI, plasma miR-133 levels increased by 4.4-fold (relative to controls) with an area under receiver operating curve (AUC) of 0.89. The study not only reported a positive correlation of elevated miR-133 with cTn but also suggested miR-133 to be superior to cTnI levels [47]. Similar results were reported in Italian patients with AMI. Plasma levels of miR-133a and miR-133b peaked at 156 ± 72 min after the onset of AMI symptoms, a time course earlier than the conventional AMI biomarker such as cTnI [48]. In a recent study, circulating levels of miR-133b were measured in plasma of post-MI patients, for one year, and they were not considered as useful predictor for left ventricular remodelling, despite their increased concentrations after MI [55]. Since the circulating levels of miR-133a increased in a time-dependent manner in the early phase of AMI and were positively correlated with cTnT in AMI patients, associating miR-133a with the occurrence and severity of coronary atherosclerosis in coronary heart disease (CHD) patients, it can be used as a predictor for diagnosing AMI and CHD [56].

Studies in animal AMI models have corroborated the findings of elevated miR-133 levels seen in patients with AMI [47, 48]. Microarray analysis in mouse model confirmed that miR-1, miR-133a, miR-208a, and miR-499 were significantly decreased in the infracted myocardium while their circulating levels were elevated, suggesting release of these miRNAs from the infarcted myocardium. This observation was well supported by significant increase in serum levels of miR-1 and miR-133a seen in patients with AMI in the same study [57]. Although miR-133 is abundantly expressed in skeletal muscles, unlike in AMI, acute hind-limb ischemia did not result in increased plasma levels of the miRNAs (miR-1 and miR-133), suggesting that the release of miRNA into the systemic circulation may possibly be tissue or miRNA specific and dependent on the extent and the timing of cell damage [48].

The miR-208 family includes 2 genes,* MIR-208a* and* MIR-208b*. miR-208a is encoded by an intron of the *α*-myosin heavy chain (MHC) gene (*MYH6*) and is exclusively expressed by cardiomyocytes [58]. In adult mice, miR-208a regulates the induction of *β*MHC expression on cardiac stress [58] which results in the concomitant upregulation of miR-208b encoded by an intron of the *β*MHC gene (*MYH7*) [59]. Deletion of miR-208a in mice prevents pathological hypertrophic remodelling and fibrosis [59]. miR-208a regulation of cardiomyocytes is thought to occur through thyroid hormone receptor-associated protein 1 (*MED13*),* MSTN*,* SOX6*,* PURB*,* SP3*, and* HP1B* genes which are negative regulators of muscle growth and hypertrophy [58, 59].

Circulating miR-208a levels were elevated only after myocardial injury in rat AMI model showing good association with cTnI [47, 60], indicating that circulating miR-208 is specific after cardiac injury. In accordance, Wang et al. reported that miR-1, miR-133a, miR-208a, and miR-499 were increased in patients with AMI. In addition, miR-208a showed highest sensitivity and specificity for AMI. An increase in miR-208a was evident in first 4 h after the onset of chest pain as compared to ~14–18 h required for cTnI peaks to occur after the onset of AMI, indicating that miRNAs are plausibly leaked into the bloodstream at an earlier stage of myocardial insult [47]. Corsten et al. replicated these findings in 32 patients with AMI as compared to controls [61]. The study reported approximately 1600-fold increase in miR-208b levels in patients with AMI. In addition, there was a good correlation between plasma miR-208b and cTnT levels, confirming the link to myocardial damage. Other similar studies failed to replicate the utility of circulating miR-208 levels in AMI patients [48, 62], maybe partly due to rapid clearance and low concentration of circulating miR-208 making it difficult to measure by TaqMan-based assays [63].

miR-499 is encoded by an intron of the* MYH7B* gene. miR-499 has been shown to be downregulated in response to hypoxia or ischemic stress in cardiomyocytes [64]. In accordance, transgenic overexpression blunts apoptosis of cardiomyocytes and infarct size following ischemia. The cardioprotective effect, at least in part, is believed to occur as a result from its targeting of the *α*- and *β*-isoforms of calcineurin and their downstream effects on dynamin-related protein 1 [64]. miR-499 is exclusively expressed in the heart [62] and was found elevated in a small study of 9 patients with ST-elevation MI (STEMI). Similar findings were reported by Wang et al. in animal model and patients with AMI [47]. The circulating levels of miR-499, like miR-208a, were below detection limit in healthy subjects [62]. In a separate study in patients with AMI, viral myocarditis, diastolic dysfunction, and acute heart failure (HF), apart from miR-208b, levels of miR-499 were also elevated in AMI and acute HF [61]. In addition, both miRNAs correlated well with circulating cTnT levels and were modestly increased in viral myocarditis and remained unchanged in diastolic dysfunction. Other miRNAs reported to be increased in AMI include miR-1291, miR-633b, miR-30c, and miR-145. Levels of miR-1291 and miR-633b showed highest sensitivity and specificity in discriminating patients from controls with an AUC of 0.91 and 0.94, respectively, whereas levels of miR-30c and miR-145 correlated with myocardial infarct size and cTnT release [65]. More importantly, the study suggested a combination of 20 miRNAs signature that may serve as a superior diagnostic biomarker for AMI. Interestingly, miR-1, miR-133a/b, miR-208a/b, and miR-499 were not among the most dysregulated miRNAs detected in this study. Of note, however, is that whole blood was used in this study which may have significantly influenced miRNA expression pattern and therefore cannot be compared directly to other studies where plasma/serum was used.

## 7. Acute Coronary Syndrome

Widera et al. performed a large population-based study on MI in 444 patients with acute coronary syndrome (ACS) [66]. The study compared levels of 6 miRNAs (miR-1, miR-133a, miR-133b, miR-208a, miR-208b, and miR-499) in patients with STEMI, non-STEMI (NSTEMI), and unstable angina. miR-208b was only detectable in NSTEMI and STEMI patients. The study confirmed the association of elevated levels of miR-208a with AMI. This was the first study to provide prognostic information in ACS given the fact that increasing levels of miR-208b were predictive of 6-month mortality and individuals with undetectable levels of miR-208b had best prognosis. The association was independent of age and gender. Plasma levels of miR-133a were higher in patients with NSTEMI or STEMI as compared to unstable angina and significantly associated with mortality risk. However, the associations were lost after adjustment for hs-TnT levels, suggesting failure of miRNA levels to improve patient outcome.

In another recent large population-based study, Devaux et al. substantiated the role of cardiac enriched miR-208b and miR-499 in well-defined patients with STEMI (*n* = 397), NSTEMI (*n* = 113), and healthy controls (*n* = 87) [67]. The study reported that both of these miRs were highly elevated (>10^5^-fold) in both STEMI and NSTEMI and nearly undetectable in healthy controls. In addition, levels of both miRNAs correlated with peak concentration of CK and cTnT. Overall, miR-499 and hs-cTnT provided comparable diagnostic value with an AUC of 0.97. Unfortunately, their combined determination did not improve the diagnostic value of single biomarkers.

In an important study, Olivieri et al. investigated whether or not circulating miRNAs could serve as a marker of NSTEMI in geriatric patients. Elderly patients frequently present with atypical NSTEMI symptoms making diagnosis of AMI difficult. The study reported that circulating levels of miR-1, -21, -133a, -423-5p, and -499-5p were significantly increased in NSTEMI patients. Importantly, the levels of miR-21 and -499-5p were elevated in NSTEMI (*n* = 31) versus acute decompensated heart failure without AMI (*n* = 32) and the diagnostic accuracy of miR-499-5p was higher than cTnT and hs-cTnT in differentiating the two groups of patients (miR-499-5p AUC = 0.86 versus cTnT AUC = 0.68 and versus hs-cTnT AUC = 0.70) [68]. miR-21 is the most significantly upregulated miRNA in cardiac disease and has been shown to contribute to myocardial disease by an effect in cardiac fibroblast [69, 70]. miR-21 levels were found to increase in cardiac fibroblasts of the failing heart as a result of increased activation of ERK-MAP kinase signaling cascade through inhibition of Sprouty 1 (SPRY1) and pharmacological inhibition of miR-21 resulted in antihypertrophic and antifibrotic effects [69].

## 8. Heart Failure

Corsten et al. reported 2-fold elevation of miR-499 in patients with acute heart failure (HF) [61]. However, other studies failed to replicate this association [62, 71]. miR-133 levels, although not significantly associated with the disease, were positively correlated with NT-proBNP [61].

Tijsen et al. performed plasma miRNA profiling in 30 patients with HF and 20 patients with dyspnoea (non-HF) to identify miR-423-5p and several additional miRs (e.g., miR-18b^*^, miR-129-5p) that might be potential markers for HF. miR-423-5p levels were found to discriminate patients with other forms of dyspnoea not related to HF. miR-423-5p was significant predictor of HF independent of age and gender. The AUC was 0.91 suggesting that this miRNA may be a useful diagnostic predictor of HF with high sensitivity and specificity. In recent studies, plasma miR-423-5p levels were significantly increased in patients with acute MI before PCI reaching baseline within 6 h [72] and elevated plasma levels of miR-423-5p positively correlated with NT-proBNP levels in patients with heart failure caused by dilated cardiomyopathy [73]. However, larger clinical studies are needed to determine clinical value of miR-423-5p as a biomarker in HF [74].

miR-126 is an endothelial specific miR encoded within an intron in* Egfl7* and is essential for ischemic-induced angiogenesis [75]. A study in patients with ischemic HF showed lower levels of circulating endothelial-enriched miR-126 [71]. The levels were negatively correlated with serum BNP levels and clinical improvements. Delivery of apoptotic bodies enriched in miR-126 triggered the CXCL12-CXCR4 dependent vascular protection cascade in apolipoprotein E^−/−^ mice [27]. However, biological significance of miR-126 levels in heart failure remains unclear.

## 9. Stable Coronary Artery Disease

Cardiac muscle enriched miR-133a and miR-208a have been shown to be elevated in patients with stable CAD [63]. Interestingly, endothelial-enriched miR-126, members of the miR-17/92a cluster (miR-17, miR-20a, and miR-92a), the antisclerotic smooth muscle enriched miR-145, and inflammation associated miR-155 were significantly downregulated in the patient group, both in the discovery phase (*n* = 36) and in the validation cohort (*n* = 31). In contrast, in another recent study by Ren et al., circulating miRNA signature consisting of miR-106b/25 cluster, miR-17/92a cluster, miR-21/590-5p family, miR-126, and miR-451 was significantly upregulated in both the discovery and validation cohorts of vulnerable CAD [76]. Members of the miR-17/92a cluster affect angiogenesis either positively or negatively. Endothelial-enriched miR-92a is antiangiogenic and represses the proangiogenic integrin *α*5 (*ITGA5*) [77]. In contrast, miR-18, another member of the miR-17/92a cluster, suppresses antiangiogenic factors in tumor cells and is proangiogenic [78]. However, endothelial activation is critical to atherosclerosis; therefore, detection of endothelial miRNAs might identify patients at risk for CAD.

## 10. Current Limitations and Future Perspective

It is clearly evident from experimental studies that miRNAs are responsible for the regulation of several biological functions and alterations in cardiovascular diseases. Current data supports the concept of using circulating cardiac and skeletal muscle cell-enriched miRNA as a biomarker in CVD. They offer certain advantages over the traditional biomarkers. First, miRNA is released early after MI and is stable in circulation. Second, miRNAs can be detected with high specificity and sensitivity. Third, circulating miRNA can offer less invasive alternative to perform population-wide screening. Despite these advantages, several critical issues need to be resolved before their use in clinical laboratories.

The major drawback of using miRNAs as biomarkers for clinical diagnosis is their laborious isolation and detection procedures. Compared to the ELISA-based detection of cTn, the gold standard for diagnosis of ACS, the PCR-based methods for detection of circulating miRNAs are highly time consuming. In addition, the current technology employed to isolate and estimate levels of miRNA requires optimization and needs to be acceptably uniform to avoid interlaboratory bias and inconsistencies. The most commonly used methods include real-time PCR and microarray which can cause thermodynamic biases. Deep sequencing can be employed to profile miRNA signatures across the entire genome and overcome these biases [79]. However, some important considerations here would be the time factor, day-to-day variation, and the cost involved highlighting the need for development of miRNA detection using a nonclassical RNA-based approach for convenient and rapid application to clinical laboratories. And for these reasons, cTn is still the best biomarker in clinical practice.

Lack of acceptable reference gene(s) for data normalization is also another major limitation. Numerous endogenous miRNAs (e.g., miR-17) and small noncoding RNAs, such as RNU6b and RNU48, seem to be the most stable reference control for circulating miRNAs [80]. However, miR-17 is increased in ACS raising concerns on its utilization for normalization [57]. Alternatively, synthetic miRNAs can be used as exogenous spike-in controls for normalization [81]. Quantification of circulating miRNA can be greatly influenced by the origin of the miRNAs (e.g., microvesicles, exosomes, and PBMCs) and can be a source of inconsistent findings. It should be noted that plasma or serum miRNA may also be contaminated with miRNAs from other blood cells and would require careful interpretation. Since a biomarker for AMI should discriminate between chest pains caused by ACS or non-ACS, the use of healthy control group is debatable. It has been demonstrated in the majority of clinical studies that the discriminative power of a single miRNA is still lower compared to cTn, possibly caused by the type of study group, sample size, and use of nonstandardized methods. Interestingly, a combination of three miRNAs (miR-1, miR-499, and miR-21) showed better diagnostic performance than hs-TnT alone in patients suspected of ACS in whom clinical and ECG findings were inconclusive [82]. The study demonstrated that circulating miRNAs hold great potential for better management of suspected ACS patients.

Finally at this point, well-designed and large-scale prospective studies are required to validate the diagnostic utility of circulating miRNA in early diagnosis of CVD. The studies should evaluate the effect of confounders and other conventional biomarkers into the diagnostic and prognostic model to improve the risk stratification. Replication of findings in independent cohorts supported by experimental animal findings would minimize false-positive reports and highlight the plausible role of miRNA of interest. More importantly, efforts to better understand the biological processes controlling miRNA release and stability are needed. Although the correlation between circulating and tissue miRNA is still not clear, increasingly more data do not support the hypothesis that circulating miRNA levels reflect the specific changes that occur in the cardiac tissue, as miRNAs could also be derived from immune cells. While fully validated miRNA-based diagnostic assays are available for diseases such as lung cancer [83] future research studies are needed to establish the role of circulating miRNAs as diagnostic and prognostic biomarker in CVD.

## 11. Conclusion 

In conclusion, the potential of circulating miRNAs as blood-based biomarker in cardiovascular disease is highly promising. For example, cardiac-specific miRNAs (miR-208, miR-499, and miR-1) and stress-related miRNA (miR-21) may be potential biomarkers for acute coronary syndrome; circulating miR-423-5p is suggested as a biomarker for heart failure. These observations suggest that circulating miRNAs may not only be useful for prediction of cardiovascular events, but also serve as sensitive biomarkers for improving the diagnostic accuracy of CVD. However, the data should be interpreted with caution as most of the data comes from a rather small number of cases. It is still unclear which of the measured miRNAs and the origin may be best suited to be used as a biomarker. A panel of miRNAs that embody the complete mechanism of myocardial damage and inflammatory and reparative process may increase diagnostic power of miRNAs for early diagnosis [84]. In addition, a combined assessment of multiple circulating miRNAs together with cTn may increase specificity and sensitivity and accelerate AMI diagnosis. There is little evidence for prognostic information of circulating miRNAs in cardiac specific diseases [85]. It is a possible future direction to seek specific circulating miRNAs with potential value in diagnosis as well as prognosis of CVD. In absence of a reliable blood-based biomarker that could complement the traditional cardiovascular disease risk assessment, circulating miRNA can provide a good alternative source of biomarker. It remains to be seen whether circulating miRNAs can fulfil this role to improve risk or severity prediction.

## Figures and Tables

**Table 1:** Studies of circulating miRNAs in patients with cardiovascular diseases.

Clinical cohort number	Controls number	Source	Circulating miRNAs detected	Association/correlation	Reference
33 AMI	30	Plasma	1, 133a, 208a, 499↑	miR-208a shows highest sensitivity and specificity comparable with cTnI	[47]

33 STEMI	17	Plasma	1, 133a, 133b, 499-5p↑	Time course of upregulated miRNA associated with cTnI	[48]

31 AMI	20	Serum	1↑	Association with CK-MB levels	[50]

93 AMI	66	Plasma	1↑	Association with QRS duration	[51]

9 AMI, 5 UAP	10	Plasma	499↑ in AMI (within 48 h)	Correlation with CK-MB	[62]

32 AMI, 36 non-AMI	—	Plasma	208b, 133a, 499↑	miR-208b and miR-499 correlation with cTnT	[61]

20 AMI	20	Whole blood	30c, 145↑ 663b, 1291↓	miR-30c and miR-145 correlated with hs-cTnT	[65]

29 ACS, 42 without ACS	—	Serum	1, 133a↑	Correlation with cTnT	[57]

444 ACS	—	Plasma	1, 133a/b, 208b↑	miR-133a and miR-208b associated with risk of death	[66]

397 STEMI, 113 NSTEMI	87	Plasma	208b, 499↑	miR-499 showed diagnostic accuracy comparable with hs-cTnT	[67]

92 AMI, 81 CHF	66	Plasma	1, 21, 133a, 423-5p, 499-5p↑	miR-499 showed diagnostic accuracy comparable with hs-cTnT	[68]

33 acute HF	34	Plasma	499↑	—	[61]

30 HF, 20 dyspnoea	39	Plasma	423-5p↑	miR-423-5p is a predictor of heart failure	[74]

33 CHF	17	Plasma	miR-126↓	miR-126 negatively correlated with age, BNP, and NYHA class	[71]

31 stable CAD	14	Plasma	17, 92a, 126, 145, 155↓	Association with age, gender, and diabetes	[63]

13 UAP with CAD	13	Plasma	106b/25, 17/92a, 21/590-5p, 126, 451↑	Association with disease	[76]

↑: increased concentrations; ↓: decreased concentrations; ACS: acute coronary syndrome; AMI: acute myocardial infarction; BNP: brain natriuretic peptide; CAD: coronary artery disease; CHF: congestive heart failure; CK-MB: creatine-kinase myocardial band; cTn: cardiac troponin; HF: heart failure; hs-cTnT: high-sensitivity cardiac troponin T; NSTEMI: non-ST elevation myocardial infarction; NYHA: New York Heart Association; STEMI: ST elevation myocardial infarction; UAP: unstable angina pectoris.

## References

[1] Rosamond W., Flegal K., Furie K. (2008). Heart disease and stroke statistics—2008 update: a report from the American Heart Association Statistics Committee and Stroke Statistics Subcommittee. *Circulation*.

[2] Lloyd-Jones D., Adams R. J., Brown T. M. (2010). Executive summary: heart disease and stroke statistics—2010 update: a report from the American Heart Association. *Circulation*.

[3] Pearson T. A., Mensah G. A., Alexander R. W. (2003). Markers of inflammation and cardiovascular disease: application to clinical and public health practice: A statement for healthcare professionals from the centers for disease control and prevention and the American Heart Association. *Circulation*.

[4] Saenger A. K., Jaffe A. S. (2008). Requiem for a heavyweight: the demise of creatine kinase-MB. *Circulation*.

[5] Vasile V. C., Babuin L., Giannitsis E., Katus H. A., Jaffe A. S. (2008). Relationship of MRI-determined infarct size and cTnI measurements in patients with ST-elevation myocardial infarction. *Clinical Chemistry*.

[6] Daniels L. B., Laughlin G. A., Clopton P., Maisel A. S., Barrett-Connor E. (2008). Barrett-Connor, Minimally elevated cardiac troponin T and elevated N-terminal pro-B-type natriuretic peptide predict mortality in older adults: results from the Rancho Bernardo Study. *Journal of the American College of Cardiology*.

[7] Sanchis J., Bardají A., Bosch X. (2012). Usefulness of high-sensitivity troponin T for the evaluation of patients with acute chest pain and no or minimal myocardial damage. *American Heart Journal*.

[8] van de Werf F., Bax J., Betriu A. (2008). Management of acute myocardial infarction in patients presenting with persistent ST-segment elevation: the Task Force on the Management of ST-Segment Elevation Acute Myocardial Infarction of the European Society of Cardiology. *European Heart Journal*.

[9] He L., Hannon G. J. (2004). MicroRNAs: small RNAs with a big role in gene regulation. *Nature Reviews Genetics*.

[10] Lee R. C., Feinbaum R. L., Ambros V. (1993). The C. elegans heterochronic gene *lin*-4 encodes small RNAs with antisense complementarity to *lin*-14. *Cell*.

[11] Friedman R. C., Farh K. K.-H., Burge C. B., Bartel D. P. (2009). Most mammalian mRNAs are conserved targets of microRNAs. *Genome Research*.

[12] van Rooij E., Liu N., Olson E. N. (2008). MicroRNAs flex their muscles. *Trends in Genetics*.

[13] Bartel D. P., Chen C.-Z. (2004). Micromanagers of gene expression: the potentially widespread influence of metazoan microRNAs. *Nature Reviews Genetics*.

[14] Stefani G., Slack F. J. (2008). Small non-coding RNAs in animal development. *Nature Reviews Molecular Cell Biology*.

[15] Krützfeldt J., Poy M. N., Stoffel M. (2006). Strategies to determine the biological function of microRNAs. *Nature Genetics*.

[16] Kozomara A., Griffiths-Jones S. (2011). MiRBase: integrating microRNA annotation and deep-sequencing data. *Nucleic Acids Research*.

[17] Lee R. C., Ambros V. (2001). An extensive class of small RNAs in *Caenorhabditis elegans*. *Science*.

[18] Aravin A. A., Lagos-Quintana M., Yalcin A. (2003). The small RNA profile during *Drosophila melanogaster* development. *Developmental Cell*.

[19] Han J., Lee Y., Yeom K.-H., Kim Y.-K., Jin H., Kim V. N. (2004). The Drosha-DGCR8 complex in primary microRNA processing. *Genes and Development*.

[20] Yi R., Qin Y., Macara I. G., Cullen B. R. (2003). Exportin-5 mediates the nuclear export of pre-microRNAs and short hairpin RNAs. *Genes and Development*.

[21] Ketting R. F. (2011). MicroRNA biogenesis and function: an overview. *Advances in Experimental Medicine and Biology*.

[22] Lim L. P., Lau N. C., Garrett-Engele P. (2005). Microarray analysis shows that some microRNAs downregulate large numbers of-target mRNAs. *Nature*.

[23] Pillai R. S., Bhattacharyya S. N., Artus C. G. (2005). Molecular biology: inhibition of translational initiation by let-7 microRNA in human cells. *Science*.

[24] Chen X., Ba Y., Ma L. (2008). Characterization of microRNAs in serum: a novel class of biomarkers for diagnosis of cancer and other diseases. *Cell Research*.

[25] Weber J. A., Baxter D. H., Zhang S. (2010). The microRNA spectrum in 12 body fluids. *Clinical Chemistry*.

[26] Kosaka N., Iguchi H., Yoshioka Y., Takeshita F., Matsuki Y., Ochiya T. (2010). Secretory mechanisms and intercellular transfer of microRNAs in living cells. *Journal of Biological Chemistry*.

[27] Zernecke A., Bidzhekov K., Noels H. (2009). Delivery of microRNA-126 by apoptotic bodies induces CXCL12-dependent vascular protection. *Science Signaling*.

[28] Hunter M. P., Ismail N., Zhang X. (2008). Detection of microRNA expression in human peripheral blood microvesicles. *PLoS ONE*.

[29] Zhang Y., Liu D., Chen X. (2010). Secreted monocytic miR-150 enhances targeted endothelial cell migration. *Molecular Cell*.

[30] Wang K., Zhang S., Weber J., Baxter D., Galas D. J. (2010). Export of microRNAs and microRNA-protective protein by mammalian cells. *Nucleic Acids Research*.

[31] Arroyo J. D., Chevillet J. R., Kroh E. M. (2011). Argonaute2 complexes carry a population of circulating microRNAs independent of vesicles in human plasma. *Proceedings of the National Academy of Sciences of the United States of America*.

[32] Vickers K. C., Palmisano B. T., Shoucri B. M., Shamburek R. D., Remaley A. T. (2011). MicroRNAs are transported in plasma and delivered to recipient cells by high-density lipoproteins. *Nature Cell Biology*.

[33] Valadi H., Ekström K., Bossios A., Sjöstrand M., Lee J. J., Lötvall J. O. (2007). Exosome-mediated transfer of mRNAs and microRNAs is a novel mechanism of genetic exchange between cells. *Nature Cell Biology*.

[34] Turchinovich A., Samatov T. R., Tonevitsky A. G., Burwinkel B. (2013). Circulating miRNAs: cell-cell communication function?. *Frontiers in Genetics*.

[35] Swarup V., Rajeswari M. R. (2007). Circulating (cell-free) nucleic acids—a promising, non-invasive tool for early detection of several human diseases. *FEBS Letters*.

[36] Zampetaki A., Willeit P., Drozdov I., Kiechl S., Mayr M. (2012). Profiling of circulating microRNAs: from single biomarkers to re-wired networks. *Cardiovascular Research*.

[37] Mitchell P. S., Parkin R. K., Kroh E. M. (2008). Circulating microRNAs as stable blood-based markers for cancer detection. *Proceedings of the National Academy of Sciences of the United States of America*.

[38] Mo M.-H., Chen L., Fu Y., Wang W., Fu S. W. (2012). Cell-free circulating miRNA biomarkers in cancer. *Journal of Cancer*.

[39] Fichtlscherer S., Zeiher A. M., Dimmeler S. (2011). Circulating microRNAs: biomarkers or mediators of cardiovascular diseases?. *Arteriosclerosis, Thrombosis, and Vascular Biology*.

[40] Zampetaki A., Kiechl S., Drozdov I. (2010). Plasma MicroRNA profiling reveals loss of endothelial MiR-126 and other MicroRNAs in type 2 diabetes. *Circulation Research*.

[41] Li S., Zhu J., Zhang W. (2011). Signature microRNA expression profile of essential hypertension and its novel link to human cytomegalovirus infection. *Circulation*.

[42] Small E. M., Olson E. N. (2011). Pervasive roles of microRNAs in cardiovascular biology. *Nature*.

[43] Wang Z., Luo X., Lu Y., Yang B. (2008). miRNAs at the heart of the matter. *Journal of Molecular Medicine*.

[44] Bauersachs J., Thum T. (2011). Biogenesis and regulation of cardiovascular MicroRNAs. *Circulation Research*.

[45] Zhao Y., Ransom J. F., Li A. (2007). Dysregulation of cardiogenesis, cardiac conduction, and cell cycle in mice lacking miRNA-1-2. *Cell*.

[46] Yang B., Lin H., Xiao J. (2007). The muscle-specific microRNA miR-1 regulates cardiac arrhythmogenic potential by targeting GJA1 and KCNJ2. *Nature Medicine*.

[47] Wang G.-K., Zhu J.-Q., Zhang J.-T. (2010). Circulating microRNA: a novel potential biomarker for early diagnosis of acute myocardial infarction in humans. *European Heart Journal*.

[48] D'Alessandra Y., Devanna P., Limana F. (2010). Circulating microRNAs are new and sensitive biomarkers of myocardial infarction. *European Heart Journal*.

[49] Gidlöf O., Andersson P., van der Pals J., Götberg M., Erlinge D. (2011). Cardiospecific microRNA plasma levels correlate with troponin and cardiac function in patients with ST elevation myocardial infarction, are selectively dependent on renal elimination, and can be detected in urine samples. *Cardiology*.

[50] Cheng Y., Tan N., Yang J. (2010). A translational study of circulating cell-free microRNA-1 in acute myocardial infarction. *Clinical Science*.

[51] Ai J., Zhang R., Li Y. (2010). Circulating microRNA-1 as a potential novel biomarker for acute myocardial infarction. *Biochemical and Biophysical Research Communications*.

[52] Han M., Toli J., Abdellatif M. (2011). MicroRNAs in the cardiovascular system. *Current Opinion in Cardiology*.

[53] Liu N., Bezprozvannaya S., Williams A. H. (2008). microRNA-133a regulates cardiomyocyte proliferation and suppresses smooth muscle gene expression in the heart. *Genes and Development*.

[54] Torella D., Iaconetti C., Catalucci D. (2011). MicroRNA-133 controls vascular smooth muscle cell phenotypic switch in vitro and vascular remodeling *in vivo*. *Circulation Research*.

[55] Bauters C., Kumarswamy R., Holzmann A. (2013). Circulating miR-133a and miR-423-5p fail as biomarkers for left ventricular remodeling after myocardial infarction. *International Journal of Cardiology*.

[56] Wang F., Long G., Zhao C. (2013). Plasma microRNA-133a is a new marker for both acute myocardial infarction and underlying coronary artery stenosis. *Journal of Translational Medicine*.

[57] Kuwabara Y., Ono K., Horie T. (2011). Increased microRNA-1 and microRNA-133a levels in serum of patients with cardiovascular disease indicate myocardial damage. *Circulation: Cardiovascular Genetics*.

[58] van Rooij E., Sutherland L. B., Qi X., Richardson J. A., Hill J., Olson E. N. (2007). Control of stress-dependent cardiac growth and gene expression by a microRNA. *Science*.

[59] Callis T. E., Pandya K., Hee Y. S. (2009). MicroRNA-208a is a regulator of cardiac hypertrophy and conduction in mice. *Journal of Clinical Investigation*.

[60] Ji X., Takahashi R., Hiura Y., Hirokawa G., Fukushima Y., Iwai N. (2009). Plasma miR-208 as a biomarker of myocardial injury. *Clinical Chemistry*.

[61] Corsten M. F., Dennert R., Jochems S. (2010). Circulating MicroRNA-208b and MicroRNA-499 reflect myocardial damage in cardiovascular disease. *Circulation: Cardiovascular Genetics*.

[62] Adachi T., Nakanishi M., Otsuka Y. (2010). Plasma microRNA 499 as a biomarker of acute myocardial infarction. *Clinical Chemistry*.

[63] Fichtlscherer S., de Rosa S., Fox H. (2010). Circulating microRNAs in patients with coronary artery disease. *Circulation Research*.

[64] Wang J.-X., Jiao J.-Q., Li Q. (2011). miR-499 regulates mitochondrial dynamics by targeting calcineurin and dynamin-related protein-1. *Nature Medicine*.

[65] Meder B., Keller A., Vogel B. (2011). MicroRNA signatures in total peripheral blood as novel biomarkers for acute myocardial infarction. *Basic Research in Cardiology*.

[66] Widera C., Gupta S. K., Lorenzen J. M. (2011). Diagnostic and prognostic impact of six circulating microRNAs in acute coronary syndrome. *Journal of Molecular and Cellular Cardiology*.

[67] Devaux Y., Vausort M., Goretti E. (2012). Use of circulating microRNAs to diagnose acute myocardial infarction. *Clinical Chemistry*.

[68] Olivieri F., Antonicelli R., Lorenzi M. (2013). Diagnostic potential of circulating miR-499-5p in elderly patients with acute non ST-elevation myocardial infarction. *International Journal of Cardiology*.

[69] Thum T., Gross C., Fiedler J. (2008). MicroRNA-21 contributes to myocardial disease by stimulating MAP kinase signalling in fibroblasts. *Nature*.

[70] Van Rooij E., Sutherland L. B., Liu N. (2006). A signature pattern of stress-responsive microRNAs that can evoke cardiac hypertrophy and heart failure. *Proceedings of the National Academy of Sciences of the United States of America*.

[71] Fukushima Y., Nakanishi M., Nonogi H., Goto Y., Iwai N. (2011). Assessment of plasma miRNAs in congestive heart failure. *Circulation Journal*.

[72] Nabiałek E., Wańha W., Kula D. (2013). Circulating microRNAs (miR423-5p, miR-208a and miR-1) in acute myocardial infarction and stable coronary heart disease. *Minerva Cardioangiologica*.

[73] Fan K.-L., Zhang H.-F., Shen J., Zhang Q., Li X.-L. (2013). Circulating microRNAs levels in Chinese heart failure patients caused by dilated cardiomyopathy. *Indian Heart Journal*.

[74] Tijsen A. J., Creemers E. E., Moerland P. D. (2010). MiR423-5p as a circulating biomarker for heart failure. *Circulation Research*.

[75] Wang S., Aurora A. B., Johnson B. A. (2008). The endothelial-specific microRNA miR-126 governs vascular integrity and angiogenesis. *Developmental Cell*.

[76] Ren J., Zhang J., Xu N. (2013). Signature of circulating MicroRNAs As potential biomarkers in vulnerable coronary artery disease. *PLoS ONE*.

[77] Bonauer A., Carmona G., Iwasaki M. (2009). MicroRNA-92a controls angiogenesis and functional recovery of ischemic tissues in Mice. *Science*.

[78] Suárez Y., Fernández-Hernando C., Yu J. (2008). Dicer-dependent endothelial microRNAs are necessary for postnatal angiogenesis. *Proceedings of the National Academy of Sciences of the United States of America*.

[79] Hu Z., Chen X., Zhao Y. (2010). Serum microRNA signatures identified in a genome-wide serum microRNA expression profiling predict survival of non-small-cell lung cancer. *Journal of Clinical Oncology*.

[80] Lai C.-Y., Yu S.-L., Hsieh M. H. (2011). MicroRNA expression aberration as potential peripheral blood biomarkers for Schizophrenia. *PLoS ONE*.

[81] Kroh E. M., Parkin R. K., Mitchell P. S., Tewari M. (2010). Analysis of circulating microRNA biomarkers in plasma and serum using quantitative reverse transcription-PCR (qRT-PCR). *Methods*.

[82] Oerlemans M. I. F. J., Mosterd A., Dekker M. S. (2012). Early assessment of acute coronary syndromes in the emergency department: the potential diagnostic value of circulating microRNAs. *EMBO Molecular Medicine*.

[83] Bianchi F., Nicassio F., Marzi M. (2011). A serum circulating miRNA diagnostic test to identify asymptomatic high-risk individuals with early stage lung cancer. *EMBO Molecular Medicine*.

[84] Li C., Fang Z., Jiang T. (2013). Serum microRNAs profile from genome-wide serves as a fingerprint for diagnosis of acute myocardial infarction and angina pectoris. *BMC Medical Genomics*.

[85] Zampetaki A., Willeit P., Tilling L. (2012). Prospective study on circulating microRNAs and risk of myocardial infarction. *Journal of the American College of Cardiology*.

